# Serum trimethylamine N-oxide and its precursors are associated with the occurrence of mild cognition impairment as well as changes in neurocognitive status

**DOI:** 10.3389/fnut.2024.1461942

**Published:** 2024-11-28

**Authors:** He Bai, Yao Zhang, Peiying Tian, Yani Wu, Ruiheng Peng, Bin Liang, Wenli Ruan, Enmao Cai, Ying Lu, Mingfeng Ma, Liqiang Zheng

**Affiliations:** ^1^School of Public Health, Shanghai Jiao Tong University School of Medicine, Shanghai, China; ^2^Department of Endocrinology, Shengjing Hospital of China Medical University, Shenyang, China; ^3^Department of Gastroenterology, Shanghai Pudong Hospital, Fudan University Pudong Medical Center, Shanghai, China; ^4^Department of Cardiovascular Medicine, Second Hospital of Shanxi Medical University, Taiyuan, China; ^5^Department of Physical and Chemical, Changning District Center for Disease Control and Prevention, Shanghai, China; ^6^Department of Cardiovascular Medicine, Fenyang Hospital of Shanxi Province, Fenyang, China; ^7^Ministry of Education-Shanghai Key Laboratory of Children's Environmental Health, Xinhua Hospital Affiliated to Shanghai Jiao Tong University School of Medicine, Shanghai, China

**Keywords:** trimethylamine N-oxide, TMAO, mild cognition impairment, MCI, MoCA-BC, choline, betaine, carnitine

## Abstract

**Background:**

This study aims to examine the association between gut microbe-dependent trimethylamine N-oxide (TMAO) and its precursors (choline, betaine, and carnitine) levels and mild cognition impairment (MCI), alongside changes in the Chinese version of the Montreal Cognitive Assessment-Basic (ΔMoCA-BC) score in rural adults.

**Methods:**

Drawing data from a large-scale epidemiological study conducted in rural areas of Fuxin County, Liaoning Province, China. 1,535 participants free from brain-related ailments were initially surveyed. MCI was assessed through the MoCA-BC score. Logistic regression models and restricted cubic spline were used to investigate the association between TMAO and its precursors levels and MCI. Additionally, the association between TMAO and its precursors levels and ΔMoCA-BC was analyzed using a generalized linear model in the longitudinal study.

**Results:**

The average age of the study participants was 58.6 ± 9.4 years and the prevalence rate of MCI was 34.5%. With the second quartile as the reference in the logistic regression model, the OR for risk of MCI in the highest quartile for TMAO, betaine, and carnitine was 1.685 (95% CI: 1.232–2.303, *p* = 0.001), 2.367 (95% CI: 1.722–3.255, *p* < 0.001), and 2.239 (95% CI: 1.742–3.295, *p* < 0.001), respectively. The OR of choline for the highest versus lowest quartile was 2.711 (95% CI: 2.012–3.817, *p* < 0.001) for the risk of MCI. We find a J-shaped association between betaine (*P*_non-linear_ = 0.001) and carnitine (*P*_non-linear_ = 0.003) levels and MCI. Furthermore, TMAO and its precursors levels were associated with ΔMoCA-BC in the third and fourth quartiles group (All *p* < 0.05).

**Conclusion:**

The findings suggest the existence of an optimal concentration range for serum levels of TMAO, betaine, and carnitine that mitigates MCI risk, paving the way for enhanced dietary interventions aimed at preventing and treating MCI.

## 1 Introduction

Dementia represents a leading cause of disability among individuals aged 65 and older globally (1). In China, approximately 15.07 million individuals aged 60 and above are affected by dementia, which constitutes about 25.5% of the worldwide dementia population (2). Currently, there is no specific pharmacological treatment for dementia (3), underscoring the importance of early prevention and intervention. Mild cognitive impairment (MCI) is recognized as a transitional phase between normal cognitive aging and dementia, placing individuals in this category at a heightened risk for developing dementia (4, 5). Research indicates that the annual conversion rate from MCI to dementia is tenfold higher than that of cognitively normal individuals (4–6). Extensive population surveys have reported a concerning MCI prevalence rate of 15.5% in China, equating to approximately 38.77 million affected individuals (4). Traditional risk factors for MCI, including a family history of dementia, hypertension, and diabetes, account for only a portion of MCI cases (7, 8). Therefore, it is crucial to identify additional factors that may influence the onset of MCI.

Trimethylamine N-oxide (TMAO) is a bioactive compound primarily synthesized through the metabolic activities of gut microbiota in the human body (9). Mounting evidence has linked TMAO and its precursors to neurocognitive disorders, notably MCI (10, 11). Through the enzymatic processing of dietary components such as carnitine, choline (including choline-containing compounds like phosphatidylcholine), and betaine (a product of choline metabolism pathway), the intestinal microbiome generates trimethylamine (TMA) within the intestinal lumen (12–14), subsequently, TMA undergoes absorption within the intestines and is ferried to the liver via the portal venous circulation, where flavin-containing monooxygenase subtypes 3 (FMO3) catalyze its oxidation into TMAO (15, 16).

In recent years, research has suggested that elevated plasma TMAO levels may infiltrate the central nervous system, triggering neuroinflammation and immune responses, and impairing the integrity of the blood–brain barrier (17). However, the findings of published studies are currently inconsistent and the underlying mechanisms are still unclear (18–21). With limited studies conducted in China, where TMAO and its precursors are influenced by diverse factors including geographical variations, dietary habits, and gut microbiota composition, our study aims to explore the association between serum TMAO and its precursors with MCI, and further investigate their potential impact on the dynamic changes in MoCA-BC score. This study not only supplements evidence from large-scale population studies in rural China of the relationship between TMAO and its precursors and MCI but also holds promise for future MCI prevention and management strategies through dietary modulation of TMAO and its precursors.

## 2 Materials and methods

### 2.1 Study population

We derived the data from a large-scale epidemiological study in the rural areas of Fuxin County, Liaoning Province, China. The baseline survey was conducted from June to August 2019. The selection of natural villages, questionnaire survey, physical examinations, and other information for this study can all be referenced from the previously published literature by our research group (22–24). Participants were eligible if: (1) they were 35 years of age or older; (2) residency in the study area for a minimum of 5 years; (3) they were willing to sign informed consent to participate in the study. Participants who were pregnant, suffered from severe liver or kidney dysfunction, or were unwilling to participate in this study were excluded (22). Finally, 4,689 participants were recruited as the study population. The follow-up survey was conducted between June and August 2021, adhering to the same inclusion and exclusion criteria as established during the baseline survey. Written informed consent was obtained from all participants. This study was approved by the human experimentation committee of China Medical University [(2018)083] (23, 24).

**Figure 1:**
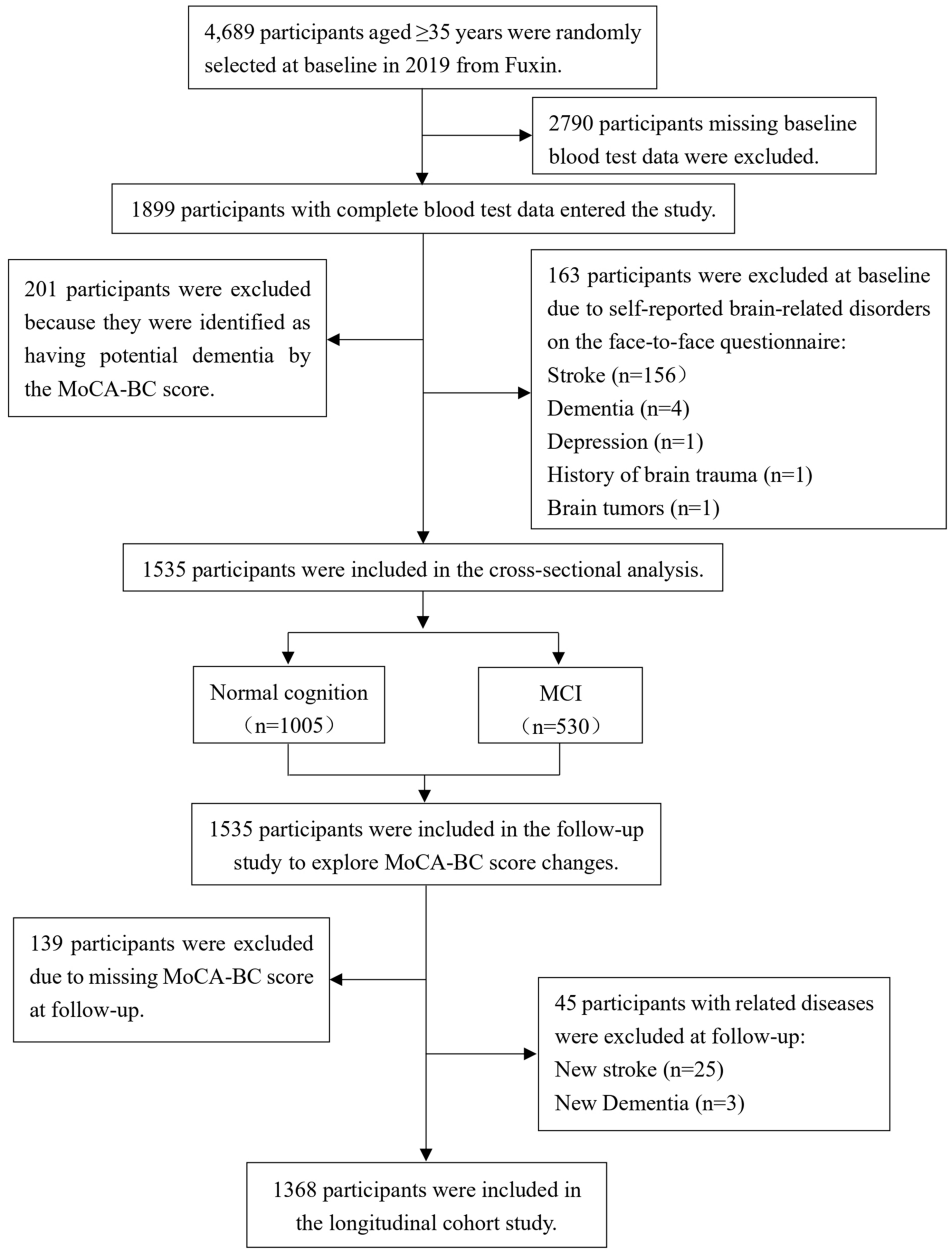
Flow diagram for the inclusion/exclusion of participants. TMAO, trimethylamine N-oxide; MCI, mild cognitive impairment; MoCA-BC, the Chinese version of the Montreal Cognitive Assessment-Basic.

The flowchart detailing the inclusion and exclusion criteria for this study is presented in Figure 1. Out of a total of 4,689 participants, 2,790 were excluded due to the absence of data on TMAO and its precursors. Additionally, 163 participants were excluded based on self-reported histories of stroke, dementia, depression, brain trauma, and brain tumors, while 201 individuals diagnosed with potential dementia were also excluded. Consequently, 1,535 participants were retained for the cross-sectional study. To further assess changes in cognitive function scores among the participants, 139 individuals lacking MoCA-BC scores were excluded during the follow-up phase, along with 28 participants who developed new cases of stroke and dementia. Ultimately, 1,368 participants were included in the longitudinal study.

### 2.2 Measurements of TMAO and its precursors

High-performance liquid chromatography–tandem mass spectrometry (HPLC-MS/MS, Shimadzu, Japan) was employed to quantify serum levels of TMAO and its precursors. Fasting venous blood samples were collected from subjects and subsequently centrifuged at 3000 rpm for 10 min. A volume of 500 μL of the upper serum layer was extracted and stored at −80°C. For the analytical procedure, 10 μL of serum was diluted 50-fold with a standard solution, vortexed for 3 min to ensure thorough mixing, and then centrifuged at 15,000 rpm for 15 min at 4°C to isolate the supernatant. The supernatant was then filtered through a 0.22 μm hydrophobic nylon membrane. Finally, 100 μL of the filtered serum was transferred into a 1.5 mL sample vial for analysis via HPLC-MS/MS.

Chromatographic separations were performed using an ACQUITY UPLC HSS T3 column (1.7 μm, 2.1 mm × 100 mm; Waters, United States). The injection volume was set at 2 μL, and the column temperature was maintained at 40°C. The mobile phases comprised phase A, consisting of 10 mmol/L ammonium formate, 0.1% formic acid, and a solvent mixture of acetonitrile and water in a 9:1 ratio, and phase B, which contained 5 mmol/L ammonium formate, 0.1% formic acid, and a solvent mixture of acetonitrile and water in a 1:1 ratio. Phase B, the aqueous component, was gradually increased over time to achieve gradient elution. Specifically, from 0.0 to 2.0 min, phase B was maintained at 10%; from 2.0 to 6.0 min, phase B was linearly increased from 10 to 45%; from 6.0 to 6.1 min, phase B was linearly increased from 45 to 100%; from 6.1 to 8.1 min, phase B was held at 100%; from 8.1 to 8.2 min, phase B was decreased from 100% back to 10%; and from 8.2 to 10.0 min, phase B was maintained at 10%. The system was equilibrated for 2.5 min, resulting in a total runtime of 10 min per sample, with a flow rate of 0.4 mL/min (split ratio 5:3).

Reference standards for each metabolite were utilized to accurately determine the chromatographic retention times and to optimize the mass spectrometry parameters. The electrospray ionization source was employed with an impact voltage of 20 eV in positive ion mode. Multiple reaction monitoring (MRM) was implemented, with operating conditions that included a drying gas flow rate of 3 L/min, an atomizer pressure of 50 psi, a drying temperature of 350°C, and a capillary voltage of 3,500 V. The linear range of detection was established between 0.16 and 20.00 μmol/L, demonstrating an excellent correlation coefficient (*r*^2^ = 0.999). Furthermore, the recovery rates for the metabolites ranged from 90.2 to 102.1%.

### 2.3 MoCA-BC test and diagnosis of MCI

The MoCA-BC is utilized as a screening instrument for MCI among elderly Chinese individuals with varying levels of educational attainment (25, 26). This assessment evaluates nine cognitive domains: executive function, language, orientation, calculation, conceptual thinking, memory, visual perception, attention, and concentration, with a maximum total score of 30 points (26). The scoring framework classifies individuals into categories of normal cognition (NC), MCI, and potential dementia, stratified by years of education (22, 27): (1) For individuals with ≤6 years of education, scores ranging from 19 to 30 indicate NC, scores from 13 to 18 suggest MCI, and scores from 0 to 12 are indicative of potential dementia; (2) For those with 7 to 12 years of education, scores of 22 to 30 denote NC, scores from 15 to 21 indicate MCI, and scores from 0 to 14 suggest potential dementia; (3) For individuals with over 12 years of education, scores ranging from 24 to 30 represent NC, scores from 16 to 23 indicate MCI, and scores from 0 to 15 are indicative of potential dementia.

In the cross-sectional study, we used the MoCA-BC to screen for NC, MCI, and potential dementia, excluding patients with potential dementia. Simultaneously, this study also focused on ΔMoCA-BC during the follow-up period, and the ΔMoCA-BC was calculated by subtracting the baseline MoCA-BC score from the follow-up MoCA-BC score.

### 2.4 Covariates

We collected information on participants’ age, gender, ethnicity, education level, family income, physical labor level, smoking, and drinking habits through face-to-face questionnaires (23). Because the local is a Mongolian autonomous county, the proportion of Mongolian is relatively high and the living habits are different from those of the Han. Therefore, the nationalities were divided into three groups: Han, Mongolian, and Others. Income was divided into three levels: less than 10,000 yuan (low-income group, lower than the average income of local residents), 10,000 to 30,000 yuan (middle-income group), and more than 30,000 yuan (high-income group, lower than the average income of local residents). Educational level was divided into three categories: primary school and below, middle school, and high school or above. Physical labor level was classified into three tiers: light, moderate, and heavy. Smokers were categorized as individuals who consumed at least one cigarette daily for a minimum of 6 months. According to the Dietary Guidelines for Chinese Residents and considering the long-term effects of alcohol consumption, drinkers were defined as individuals who consumed alcohol at least 3 times per week for at least 6 consecutive months.

In addition, Height and weight were measured using a calibrated domestic height and weight measurement instrument (Sitai Corporation, China). Body mass index (BMI) was calculated in terms of weight (kg)/height (m)^2^. Fasting blood glucose was determined using the glucose oxidase method, triglycerides (TG) were measured using colorimetric method, and total cholesterol (TC), low-density lipoprotein cholesterol (LDL-C), and high-density lipoprotein cholesterol (HDL-C) were measured using enzymatic colorimetric method. The above blood parameters were analyzed using the Cobas 8,000 C701 fully automated biochemical analyzer (Roche, Switzerland). Blood pressure was measured using the HEM-8102A/K electronic blood pressure monitor (Omron Corporation, Japan). Three measurements were taken, with an interval of more than 1 min between each measurement, the systolic blood pressure (SBP) and diastolic blood pressure (DBP) were calculated as the average of the three measurements.

### 2.5 Statistical analysis

Both TMAO and its precursors were converted into categorical variables by the interquartile range and grouped as follows: TMAO (Q1: < 2.24 μmol/L, Q2: 2.24–4.26 μmol/L, Q3: 4.27–7.75 μmol/L, Q4: > 7.75 μmol/L), choline (Q1: < 118.11 μmol/L, Q2: 118.11–162.40 μmol/L, Q3: 162.41–270.17 μmol/L, Q4: > 270.17 μmol/L), betaine (Q1: < 69.24 μmol/L, Q2: 69.24–92.50 μmol/L, Q3: 92.51–119.67 μmol/L, Q4: > 119.67 μmol/L), carnitine (Q1: < 40.09 μmol/L, Q2: 40.09–51.18 μmol/L, Q3: 51.19–70.90 μmol/L, Q4: > 70.90 μmol/L).

Continuous variables are represented as the mean ± standard deviation (SD) or medians (interquartile ranges, IQR), and categorical variables are reported as frequencies (percentages). The comparison of baseline characteristics between MCI and NC groups was conducted using Student’s *t*-test, Mann–Whitney *U* test, or chi-square test. We used a binary logistic regression model to analyze the association between the levels of TMAO and its precursors and MCI, adjusting for covariates including age, sex, BMI, smoking, drinking, education level, SBP, DBP, fasting glucose, triglyceride, and total cholesterol. Odds ratios (OR) and their 95% confidence intervals (CI) were calculated. TMAO and its precursors were entered into the model as categorical variables, with Q2 (lowest prevalence) as the reference group for TMAO, betaine, and carnitine, and Q1 as the reference group for choline. Additionally, we used restricted cubic spline (RCS) based on the logistic regression model, adjusting for the same confounding factors, and using the 25th, 50th, and 75th percentiles as the nodes for the concentration levels of TMAO and its precursors. We also conducted subgroup analyses based on gender and age among the study participants. In the longitudinal study, we used generalized linear models to investigate the association between TMAO and its precursors and ΔMoCA-BC. All statistical analyses were performed using R software (version 2.2.3) and IBM SPSS statistical software (version 25.0).

## 3 Results

### 3.1 Characteristics of the study population

**Figure 2:**
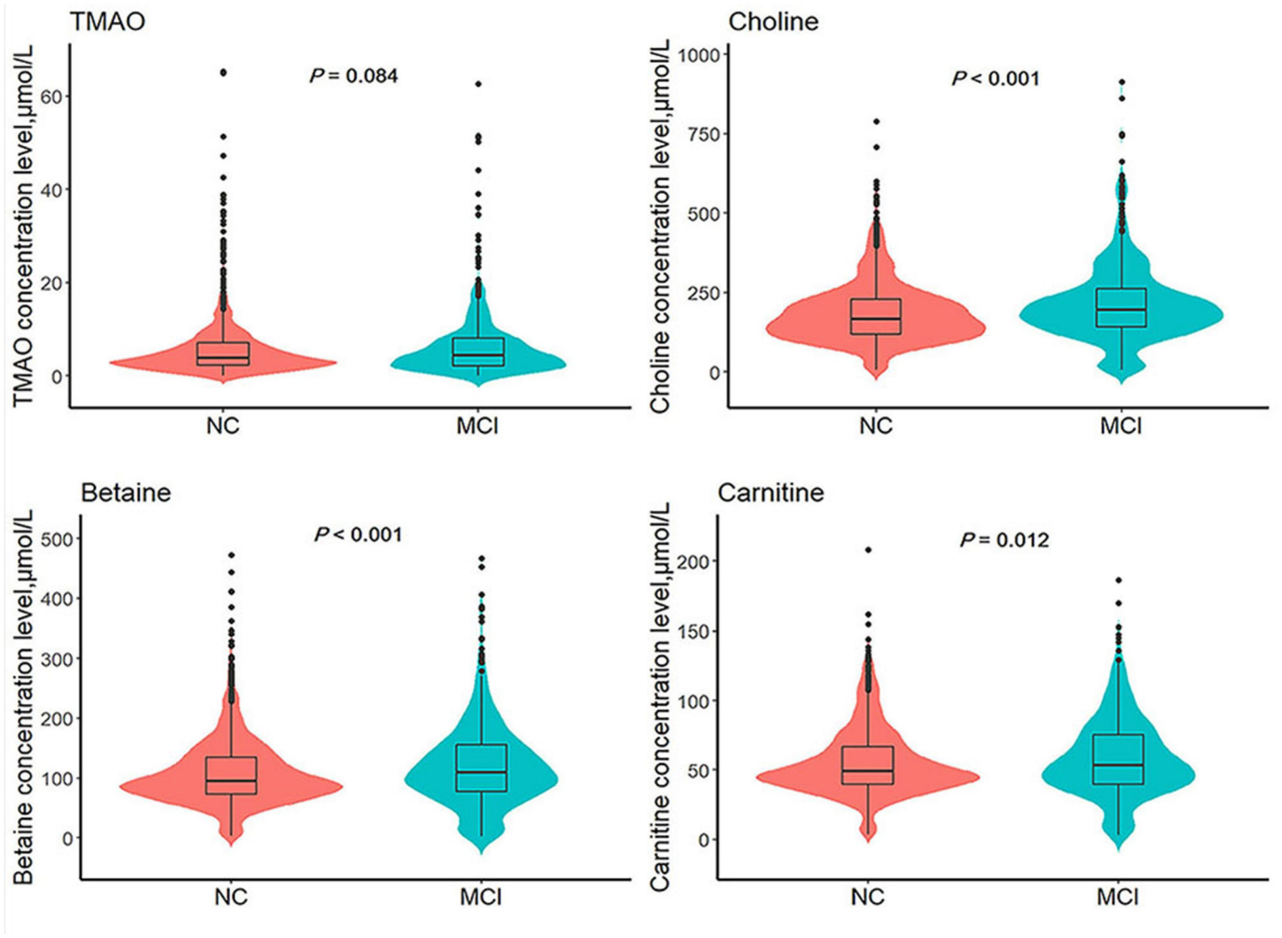
Comparison of TMAO and its precursors levels between MCI group and NC group. TMAO, trimethylamine N-oxide; NC, normal cognition; MCI, mild cognitive impairment. *p* values are calculated by Mann–Whitney *U* test.

Table 1 presents a detailed summary of the key demographic and biochemical characteristics of the study. Among the 1,535 participants at baseline, the mean age was 58.6 ± 9.4 years, with 1,038 individuals (67.6%) identified as female. A total of 530 participants were diagnosed with MCI, yielding a prevalence rate of 34.5%. Comparative analyses indicated notable differences between the MCI and NC groups, with MCI participants generally being older, having a higher proportion of females, lower BMI, reduced educational attainment, a greater prevalence of smoking, elevated fasting blood glucose levels, increased SBP, and higher total cholesterol levels. Although no significant difference in TMAO levels [median (IQR)] was observed between the NC and MCI groups [3.84 (2.24, 7.06) vs. 4.40 (2.14, 8.08), *p* = 0.084], quartile transformation revealed that TMAO levels were significantly higher in the MCI group (*p* = 0.012). In contrast, levels of choline, betaine, and carnitine were consistently higher in the MCI group, regardless of whether they were analyzed as continuous or categorical variables (see Table 1 and Figure 2).

**Table 1:** Demographic and biochemical characteristics of the participants between controls and cases of MCI at baseline.

	Total	NC	MCI	*p*
	*n* = 1,535	*n* = 1,005	*n* = 530	
Age^a^, y	58.6 ± 9.4	57.4 ± 9.7	60.8 ± 8.5	**<0.001**
Female, *n* (%)	1,038 (67.6)	708 (70.4)	330 (62.3)	**0.001**
BMI^a^, kg/m^2^	24.9 ± 3.7	25.1 ± 3.8	24.5 ± 3.5	**0.004**
Education level, *n* (%)				**0.006**
Illiterate or primary school	591 (38.5)	360 (35.8)	231 (43.6)	
Junior high school	690 (45.0)	464 (46.2)	226 (42.6)	
Tertiary high school or higher	254 (16.5)	181 (18.0)	73 (13.8)	
Ethnicity, *n* (%)				0.780
Han ethnicity	992 (64.6)	646 (64.3)	346 (65.3)	
Mongolian	487 (31.7)	320 (31.8)	167 (31.5)	
Others	56 (3.6)	39 (3.9)	17 (3.2)	
Income, *n* (%)				0.940
<10,000 yuan	1,099 (71.6)	721 (71.7)	378 (71.3)	
10,000–30,000 yuan	361 (23.5)	234 (23.3)	127 (24.0)	
≥30,000 yuan	75 (4.9)	50 (5.0)	25 (4.7)	
Physical labor level, *n* (%)				0.156
Low	421 (27.4)	263 (26.2)	158 (29.8)	
Moderate	1,050 (68.4)	695 (69.2)	355 (67.0)	
High	64 (4.2)	47 (4.7)	17 (3.2)	
Smoker, *n* (%)	496 (32.3)	302 (30.0)	194 (36.6)	**0.011**
Drinker, *n* (%)	311 (20.3)	190 (18.9)	121 (22.8)	0.080
Fasting glucose^b^, mmol/L	5.5 [5.1,6.0]	5.4 [5.1,6.0]	5.6 [5.2,6.1]	**0.001**
SBP^a^, mmHg	133.5 ± 20.3	132.4 ± 20.5	135.8 ± 20.0	**0.002**
DBP^a^, mmHg	80.3 ± 10.7	80.1 ± 10.9	80.7 ± 10.5	0.372
LDL-C^a^, mmol/L	3.3 ± 0.8	3.3 ± 0.8	3.3 ± 0.8	0.259
HDL-C^a^, mmol/L	1.2 ± 0.2	1.2 ± 0.2	1.2 ± 0.3	0.696
Triglyceride^b^, mmol/L	1.3 [0.9,1.9]	1.3 [0.9,1.8]	1.3 [0.9,2.0]	0.711
Total cholesterol^a^, mmol/L	5.2 ± 1.0	5.2 ± 0.9	5.3 ± 1.0	**0.041**
TMAO^b^, μmol/L	4.05 [2.20,7.43]	3.84 [2.24,7.06]	4.40 [2.14,8.08]	0.084
Choline^b^, μmol/L	179.12 [125.44,239.80]	167.52 [118.79,229.72]	195.34 [142.88,262.14]	**<0.001**
Betaine^b^, μmol/L	99.40 [74.26,140.99]	95.57 [73.45,134.56]	109.72 [76.96,155.72]	**<0.001**
Carnitine^b^, μmol/L	50.43 [39.91,69.81]	49.47 [39.96,66.83]	53.71 [39.78,75.39]	**0.012**
TMAO, *n* (%)				**0.012**
Q1 (<2.24 μmol/L)	394 (25.7)	253 (25.2)	141 (26.6)	
Q2 (2.24–4.26 μmol/L)	403 (26.3)	289 (28.8)	114 (21.5)	
Q3 (4.27–7.75 μmol/L)	382 (24.9)	247 (24.6)	135 (25.5)	
Q4 (>7.75 μmol/L)	356 (23.2)	216 (21.5)	140 (26.4)	
Choline, *n* (%)				**<0.001**
Q1(<118.11 μmol/L)	414 (27.0)	306 (30.4)	108 (20.4)	
Q2(118.11–162.40 μmol/L)	390 (25.4)	260 (25.9)	130 (24.5)	
Q3(162.41–270.17 μmol/L)	387 (25.2)	245 (24.4)	142 (26.8)	
Q4(>270.17 μmol/L)	344 (22.4)	194 (19.3)	150 (28.3)	
Betaine, n(%)				**<0.001**
Q1(<69.24 μmol/L)	404 (26.3)	276 (27.5)	128 (24.2)	
Q2(69.24–92.50 μmol/L)	407 (26.5)	296 (29.5)	111 (20.9)	
Q3(92.51–119.67 μmol/L)	377 (24.6)	237 (23.6)	140 (26.4)	
Q4(>119.67 μmol/L)	347 (22.6)	196 (19.5)	151 (28.5)	
Carnitine, *n* (%)				**<0.001**
Q1(<40.09 μmol/L)	392 (25.5)	257 (25.6)	135 (25.5)	
Q2(40.09–51.18 μmol/L)	408 (26.6)	300 (29.9)	108 (20.4)	
Q3(51.19–70.90 μmol/L)	367 (23.9)	231 (23.0)	136 (25.7)	
Q4(>70.90 μmol/L)	368 (24.0)	217 (21.6)	151 (28.5)	

NC, normal cognition; MCI, mild cognitive impairment; BMI, body mass index (calculated as weight in kilograms divided by height in meters squared); SBP, systolic blood pressure; DBP, diastolic blood pressure; LDL-C, low-density lipoprotein cholesterol; HDL-C, high-density lipoprotein cholesterol; TMAO, trimethylamine N-oxide; Ref, reference. Data are mean (SD), median (interquartile range), or n (%); ^a^Mean (SD). ^b^Median (interquartile range). *p* values of categorical variables are calculated by *χ*^2^ test, and continuous variables are calculated by student’s *t*-test or Mann–Whitney U test.

### 3.2 Association between TMAO and its precursors with MCI

As shown in Table 2, the prevalence of MCI exhibited a distinct trend across quartiles for TMAO, betaine, carnitine, and choline. Following adjustment for pertinent confounding variables, with Q2 serving as the reference for TMAO, participants in the Q1 group faced a 50.4% higher MCI risk (OR: 1.504, 95% CI: 1.107–2.045, *p* = 0.009). Similarly, elevated MCI risks were evident in Q3 (OR: 1.406, 95% CI: 1.031–1.917, *p* = 0.031) and Q4 (OR: 1.685, 95% CI: 1.232–2.303, *p* = 0.001) groups for TMAO. Concerning betaine and carnitine, individuals within the Q4 group exhibited a 136.7% higher risk of MCI for betaine (OR: 2.367, 95% CI: 1.722–3.255, *p* < 0.001) and a 123.9% higher risk of MCI for carnitine (OR: 2.239, 95% CI: 1.742–3.295, *p* < 0.001) compared to those in the Q2 group. The risk of MCI associated with betaine and carnitine levels corresponded consistently with that of TMAO. These findings suggest an optimal concentration range for TMAO, betaine, and carnitine in MCI prevention, with concentrations below or above this range potentially escalating MCI risk. In contrast, choline showcased a distinctive pattern, with progressively escalating MCI risk across quartiles. Compared to the lowest Q1 group, the MCI risk surged in the Q2 (OR: 1.555, 95% CI: 1.139–2.124, *p* = 0.005), Q3 (OR: 2.040, 95% CI: 1.489–2.794, *p* < 0.001), and Q4 (OR: 2.711, 95% CI: 2.012–3.817, *p* < 0.001) groups in the adjusted model.

**Table 2:** Association between serum TMAO and its precursors levels and prevalent MCI.

	Total	MCI *n* (%)	Model 1		Model 2	
			OR (95% CI)	*P _value_*	OR (95% CI)	*P _value_*
TMAO, μmol/L						
Q1(<2.24)	394	141 (35.8)	1.413 (1.048, 1.905)	**0.023**	1.504 (1.107,2.045)	**0.009**
Q2(2.24–4.26)	403	114 (28.3)	1.000 (Ref.)		1.000 (Ref.)	
Q3(4.27–7.75)	382	135 (35.3)	1.386 (1.025, 1.873)	**0.034**	1.406 (1.031,1.917)	**0.031**
Q4(>7.75)	356	140 (39.3)	1.643 (1.213, 2.226)	**0.001**	1.685 (1.232,2.303)	**0.001**
*P _trend_*				**0.012**		**0.008**
Choline, μmol/L						
Q1(<118.11)	414	108 (26.1)	1.000 (Ref.)		1.000 (Ref.)	
Q2(118.11–162.40)	390	130 (33.3)	1.417 (1.045, 1.920)	**0.025**	1.555 (1.139,2.124)	**0.005**
Q3(162.41–270.17)	387	142 (36.7)	1.642 (1.215, 2.220)	**0.001**	2.040 (1.489,2.794)	**<0.001**
Q4(>270.17)	344	150 (43.6)	2.191 (1.613, 2.974)	**<0.001**	2.711 (2.012,3.817)	**<0.001**
*P _trend_*				**<0.001**		**<0.001**
Betaine, μmol/L						
Q1(<69.24)	404	128 (31.7)	1.237 (0.914, 1.674)	0.169	1.275 (0.933,1.741)	0.127
Q2(69.24–92.50)	407	111 (27.3)	1.000 (Ref.)		1.000 (Ref.)	
Q3(92.51–119.67)	377	140 (37.1)	1.575 (1.165, 2.131)	**0.003**	1.711 (1.251,2.339)	**0.001**
Q4(>119.67)	347	151 (43.5)	2.054 (1.515, 2.785)	**<0.001**	2.367 (1.722,3.255)	**<0.001**
*P _trend_*				**<0.001**		**<0.001**
Carnitine, μmol/L						
Q1(<40.09)	392	135 (34.4)	1.459 (1.078, 1.975)	**0.015**	1.474 (1.077,2.017)	**0.015**
Q2(40.09–51.18)	408	108 (26.5)	1.000 (Ref.)		1.000 (Ref.)	
Q3(51.19–70.90)	367	136 (37.1)	1.645 (1.205, 2.219)	**0.002**	1.768 (1.289,2.425)	**<0.001**
Q4(>70.90)	368	151 (41.0)	1.933 (1.428, 2.616)	**<0.001**	2.239 (1.742,3.295)	**<0.001**
*P _trend_*				**<0.001**		**<0.001**

OR, odds ratio; CI, confidence interval; TMAO, trimethylamine N-oxide; Ref, reference; BMI, body mass index; SBP, systolic blood pressure; DBP, diastolic blood pressure; MCI, mild cognitive impairment. Model 1: unadjusted; Model 2: adjusted for age, sex, BMI, smoking, drinking, education level, SBP, DBP, fasting glucose, triglyceride, and total cholesterol. ORs and CIs were calculated using binary logistic regression models.

**Figure 3:**
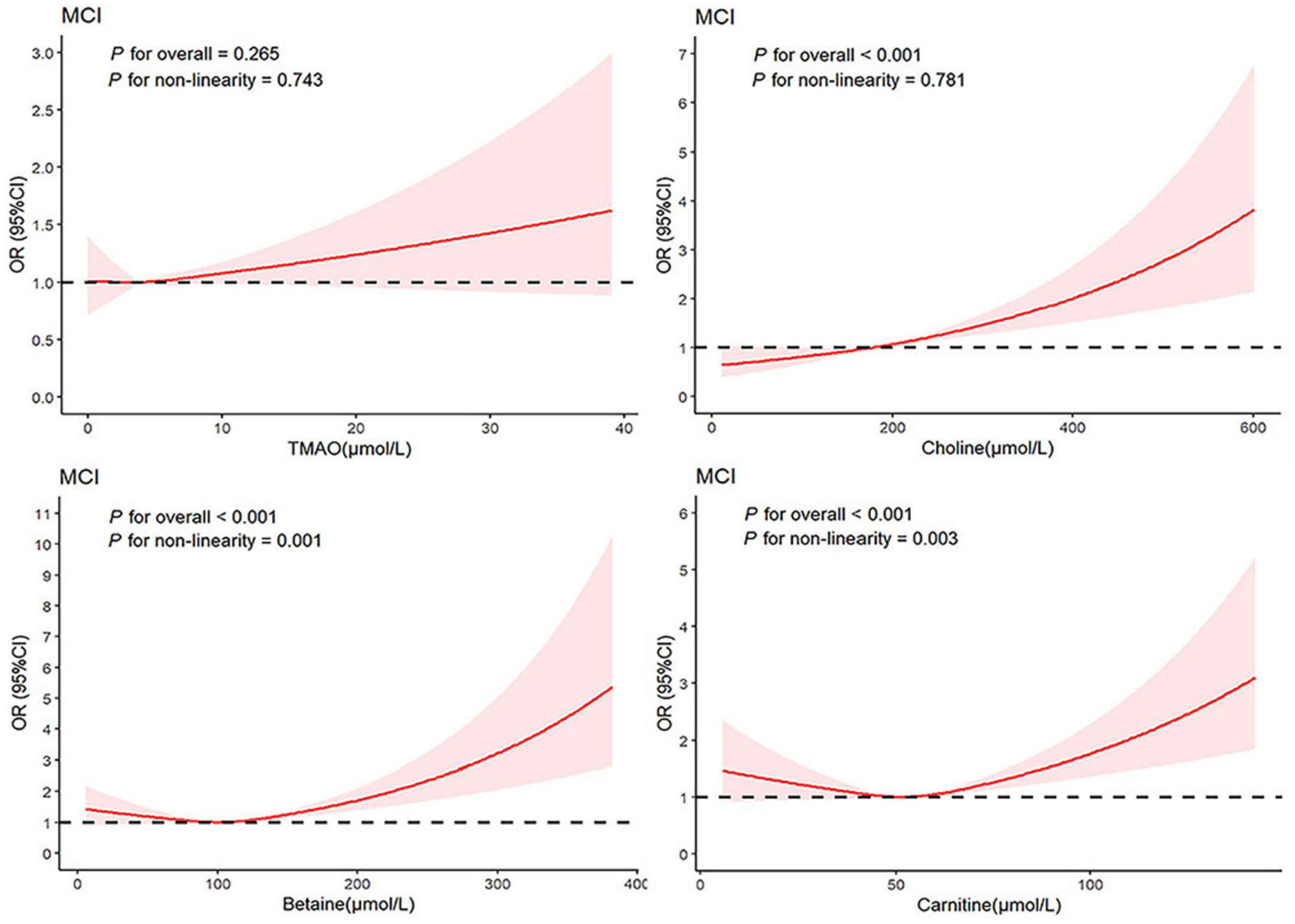
Association between TMAO and their precursors levels and MCI using a restricted cubic spline regression. Multivariable adjusted odds ratio (ORs) and 95% CIs were derived from restricted cubic spline regression, with knots placed at the 25th, 50th, and 75th percentiles of TMAO and their precursor levels. The solid red line represents multivariable-adjusted odds ratios, with the shaded part showing a 95% CI. Model adjusted for age, sex, BMI, smoking, drinking, education level, SBP, DBP, fasting glucose, triglyceride, and total cholesterol. TMAO, trimethylamine N-oxide; MCI, mild cognitive impairment; ORs, odds ratio; CIs, confidence interval.

Additionally, employing multivariable-adjusted restricted cubic spline plots (refer to Figure 3), no significant association was observed between TMAO and MCI (*P*_overall_ = 0.265). While choline exhibited a linear relationship with MCI (*P*_non-linear_ = 0.781), both betaine (*P*_non-linear_ = 0.001) and carnitine (*P*_non-linear_ = 0.003) showcased a J-shaped association with MCI, indicating complex dynamics.

### 3.3 Association between TMAO and its precursors with ΔMoCA-BC

**Figure 4:**
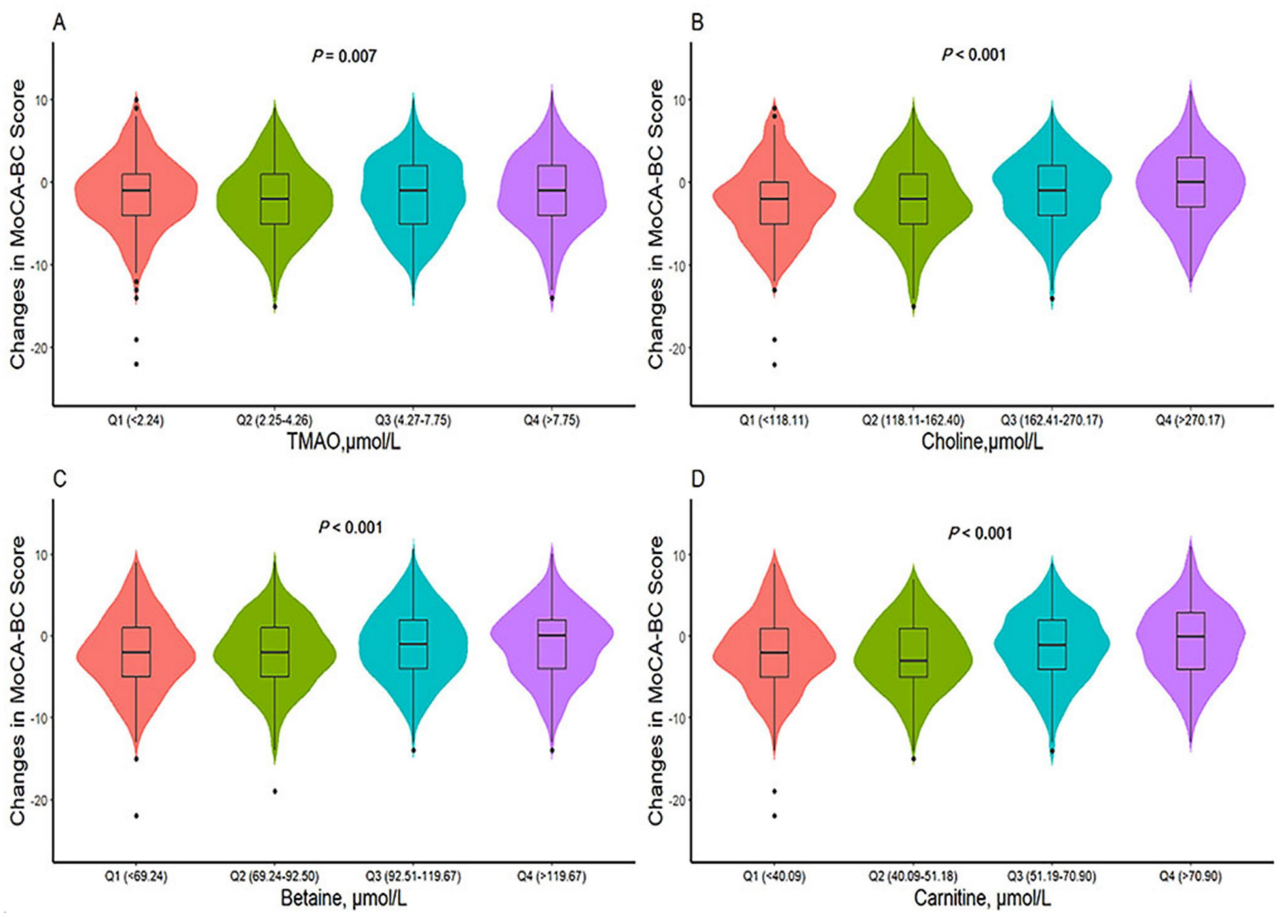
Differences in ΔMoCA-BC score of TMAO and its precursors levels among different groups. TMAO, trimethylamine N-oxide; MoCA-BC, the Chinese version of the Montreal Cognitive Assessment-Basic. *p* values are calculated by Kruskal-Wallis test. **(A)** Differences in ΔMoCA-BC score of TMAO levels among different groups. **(B)** Differences in ΔMoCA-BC score of choline levels among different groups. **(C)** Differences in ΔMoCA-BC score of betaine levels among different groups. **(D)** Differences in ΔMoCA-BC score of carnitine levels among different groups.

The ΔMoCA-BC value (mean ± SD) was −1.6 ± 4.5 points over a two-year follow-up period. Analysis presented in Figure 4 revealed significant variations in ΔMoCA-BC across different levels of TMAO, choline, betaine, and carnitine. Utilizing a generalized linear model, we examined the complex relationship between TMAO and its precursor levels and ΔMoCA-BC (see Table 3). Relative to the Q2 group, the ΔMoCA-BC demonstrated a significant increase of 0.682 in the Q1 group for TMAO levels, with similar increases of 0.679 and 0.976 observed in the Q3 and Q4 groups, respectively, all of which were statistically significant. These results highlight the intricate interplay between TMAO levels and alterations in neurocognitive function, suggesting the existence of an optimal range of TMAO levels that exerts minimal impact on ΔMoCA-BC. Furthermore, ΔMoCA-BC increased by 0.903 for betaine and 1.301 for carnitine in the Q4 group compared to the Q2 group. In contrast, choline exhibited a significant positive increase in ΔMoCA-BC of 0.777 and 1.384 for the Q3 and Q4 groups, respectively, when compared to the lowest Q1 group.

**Table 3:** Association of TMAO and its precursors levels on changes in MoCA-BC score.

	*β*	SE	95% CI	*p*
TMAO, μmol/L				
Q1 (<2.24)	0.682	0.322	(0.052, 1.313)	**0.034**
Q2 (2.24–4.26)	0.000 (Ref.)			
Q3 (4.27–7.75)	0.679	0.327	(0.039, 1.319)	**0.038**
Q4 (>7.75)	0.976	0.341	(0.299, 1.635)	**0.005**
Choline, μmol/L				
Q1 (<118.11)	0.000 (Ref.)			
Q2 (118.11–162.40)	0.088	0.320	(−0.539, 0.714)	0.784
Q3 (162.41–270.17)	0.777	0.331	(0.128, 1.426)	**0.019**
Q4 (>270.17)	1.384	0.350	(0.698, 2.070)	**<0.001**
Betaine, μmol/L				
Q1 (<69.24)	−0.009	0.319	(−0.635, 0.617)	0.978
Q2 (69.24–92.50)	0.000 (Ref.)			
Q3 (92.51–119.67)	0.932	0.329	(0.281, 1.583)	**0.005**
Q4 (>119.67)	0.903	0.346	(0.216, 1.590)	**0.010**
Carnitine, μmol/L				
Q1 (<40.09)	0.455	0.326	(−0.183, 1.093)	0.163
Q2 (40.09–51.18)	0.000 (Ref.)			
Q3 (51.19–70.90)	0.872	0.330	(0.226, 1.518)	**0.008**
Q4 (>70.90)	1.301	0.341	(0.632, 1.971)	**<0.001**

TMAO, trimethylamine N-oxide; MoCA-BC, the Chinese version of the Montreal Cognitive Assessment; SE, standard Error; CIs, confidence interval. Model adjusted for age, sex, BMI, smoking, drinking, education level, SBP, DBP, fasting glucose, triglyceride, and total cholesterol. Data were calculated using a generalized linear model.

### 3.4 Subgroup analysis

Subgroup analysis further delineated the impact of TMAO and its precursors on MCI risk (refer to Supplementary Figures S1–S4), stratified by gender (male, female) and age (< 60 years, ≥ 60 years), and no interaction was found between TMAO and its precursors and gender (*P _interaction_* > 0.05). In males, the MCI risk in the Q1 group for TMAO was higher than that in the Q2 group, with an OR of 2.069 (95% CI: 1.219–3.513), while there was no significant difference in females. Conversely, no significant difference was observed in females. These findings were consistent with the results of the unstratified analysis. Furthermore, the association between MCI risk and the levels of TMAO and its precursors was statistically significant in the cohort aged 60 years and older.

## 4 Discussion

This study demonstrates that both low and high levels of TMAO, betaine, and carnitine are associated with an elevated risk of MCI, whereas moderate concentrations (Q2 group) of these metabolites may confer protective effects. Furthermore, elevated levels of TMAO and its precursors correlate with an increase in ΔMoCA-BC score. The observed bidirectional relationship between TMAO, betaine, carnitine, and MCI risk may be influenced by various factors. The dosage of TMAO could also play a role in cognitive impairment, as typical fasting blood levels in the general population range from 3.6 to 3.7 μmol/L (28), which falls within the Q2 concentration range identified in our study. Animal studies have indicated that TMAO at physiological concentrations can significantly enhance blood–brain barrier integrity following acute inflammation in murine models, thereby improving cognitive function (29). Additionally, TMAO has been shown to mitigate neurological dysfunction in diabetic rat models (20). These findings from animal studies support the notion that TMAO may exert beneficial effects on cognitive function at specific concentration ranges. This suggests that TMAO and its precursors may provide protective effects through the activation of innate defense mechanisms or the facilitation of repair processes at optimal concentrations. Previous research has highlighted that this protective role of TMAO may be mediated through antioxidant pathways. For instance, TMAO can activate the Nrf2 signaling pathway, leading to increased expression of antioxidant genes, reduced muscle cell damage, and enhanced protection of neurons against oxidative stress (30).

Elevated concentrations of TMAO have been identified as a potential risk factor for cognitive impairment, a conclusion supported by numerous studies. Specifically, high TMAO levels serve as a significant independent risk factor for MCI in patients with type 2 diabetes (11). A cross-sectional study has demonstrated increased TMAO levels in the cerebrospinal fluid of individuals with MCI, revealing a direct correlation between TMAO concentrations and markers of cognitive dysfunction and neurodegeneration (31). Furthermore, prospective cohort studies indicate that elevated baseline plasma TMAO levels are significantly associated with cognitive dysfunction following transient ischemic attacks (32). These findings are consistent with our observations that high TMAO levels exacerbate cognitive impairment, thereby increasing the risk of MCI. Investigations into the mechanisms underlying TMAO-induced MCI suggest that elevated TMAO levels may initiate inflammatory cascades, particularly through the activation of the NF-kB pathway, which heightens the risk of neuroinflammation when the integrity of the blood–brain barrier is compromised, ultimately contributing to cognitive decline (33–35). Additionally, elevated TMAO levels may accelerate brain aging by promoting mitochondrial damage and increasing superoxide production, thereby exacerbating age-related cognitive dysfunction (19, 36, 37). Collectively, these findings underscore the detrimental impact of elevated TMAO concentrations on cognitive function and emphasize the necessity for precise regulation of TMAO and its precursors to preserve cognitive health.

We initially identified a J-shaped association between serum levels of betaine and carnitine and the risk of MCI, suggesting that deviations from optimal concentrations may elevate the risk of MCI. This observation may imply that betaine and carnitine operate through distinct metabolic pathways or signaling mechanisms at varying concentration levels. However, existing literature indicates a negative correlation between plasma betaine levels and cognitive impairment in stroke patients (38). Chen et al. reported an inverse relationship between elevated plasma carnitine levels and cognitive impairment 3 months post-stroke, which is not entirely congruent with our findings (39). Research has demonstrated that the metabolism of betaine and carnitine is significantly influenced by dietary intake and gut microbiota (16). Consequently, their serum levels and the associated relationship with cognitive function may be modulated by a variety of factors. Both excessively low and high concentrations may disrupt metabolic equilibrium and adversely affect cognitive function. Additionally, the positive correlation between choline levels and the risk of MCI was corroborated in this study, aligning with previous research that has reported elevated choline levels in patients with MCI or Alzheimer’s disease (AD) (40, 41). While some studies indicate that increased choline levels may confer protective effects on cognitive function under specific conditions, they may also exacerbate neuroinflammation and elevate the risk of MCI, particularly in the context of metabolic imbalances or dysbiosis of the gut microbiota (42, 43).

In our study, the observed average decline of 1.6 points in the MoCA-BC score during the follow-up period underscores the dynamic nature of cognitive function and emphasizes the necessity of longitudinal assessments for monitoring cognitive decline. Consistent with prior research on MCI and cerebral small vessel disease, we identified that TMAO levels outside the range of 2.24–4.26 μmol/L were associated with an increase in ΔMoCA-BC (44), suggesting that TMAO may exert a beneficial effect on cognitive function when present at concentrations outside its optimal range. Additionally, the correlation between elevated levels of choline, betaine, and carnitine and cognitive improvements further supports the potential influence of these metabolites on cognitive function. However, the variability in findings from prospective cohort studies may be attributable to differences in baseline MoCA-BC scores and shorter follow-up durations, necessitating careful interpretation of our results.

This study, conducted on a large cohort from rural Northeast China, provides comprehensive measurements of serum concentrations of choline, betaine, and carnitine, thereby contributing to the population-based evidence regarding the association between TMAO precursors and MCI. It represents the first longitudinal investigation into the relationship between TMAO and its precursors with changes in ΔMoCA-BC. However, several limitations should be acknowledged. Firstly, the relatively small sample size and the brief follow-up period may hinder the assessment of the long-term effects of TMAO and its precursors on cognitive function. Secondly, the exclusive reliance on the MoCA-BC score for defining MCI may have led to an inflated prevalence rate while compromising specificity. Additionally, the regional and lifestyle characteristics of the sample may restrict the generalizability of the findings. Future research should focus on validating these findings in larger and more diverse populations, as well as expanding animal studies to elucidate the mechanistic pathways linking TMAO, betaine, carnitine, and choline to MCI. Such investigations will contribute to improved strategies for the prevention and management of MCI.

## 5 Conclusion

In a large-sample population study conducted in rural areas of Liaoning Province, we found that serum levels of TMAO, choline, betaine, and carnitine are not only associated with MCI but also correlated with changes in ΔMoCA-BC. Our analysis revealed a J-shaped relationship between betaine and carnitine levels and the incidence of MCI, suggesting the existence of an optimal concentration range for serum levels of TMAO, betaine, and carnitine that may minimize the risk of MCI. Furthermore, elevated levels of TMAO and its precursors were linked to an increase in ΔMoCA-BC. Future research should focus on the dynamic changes in neurocognitive function, and dietary guidance may play a crucial role in the prevention and management of MCI.

## Data Availability

The original contributions presented in the study are included in the article/Supplementary material, further inquiries can be directed to the corresponding author.
