# Supplementary material for: Serum trimethylamine N-oxide and its precursors are associated with the occurrence of mild cognition impairment as well as changes in neurocognitive status
Source: Front Nutr. 2024 Nov 28;11:1461942. doi: 10.3389/fnut.2024.1461942 (PMC11634597; doi:10.3389/fnut.2024.1461942)
Supplement: Supplementary file 1 [file Image_1.pdf]

Supplementary Material

1 Supplementary Figures

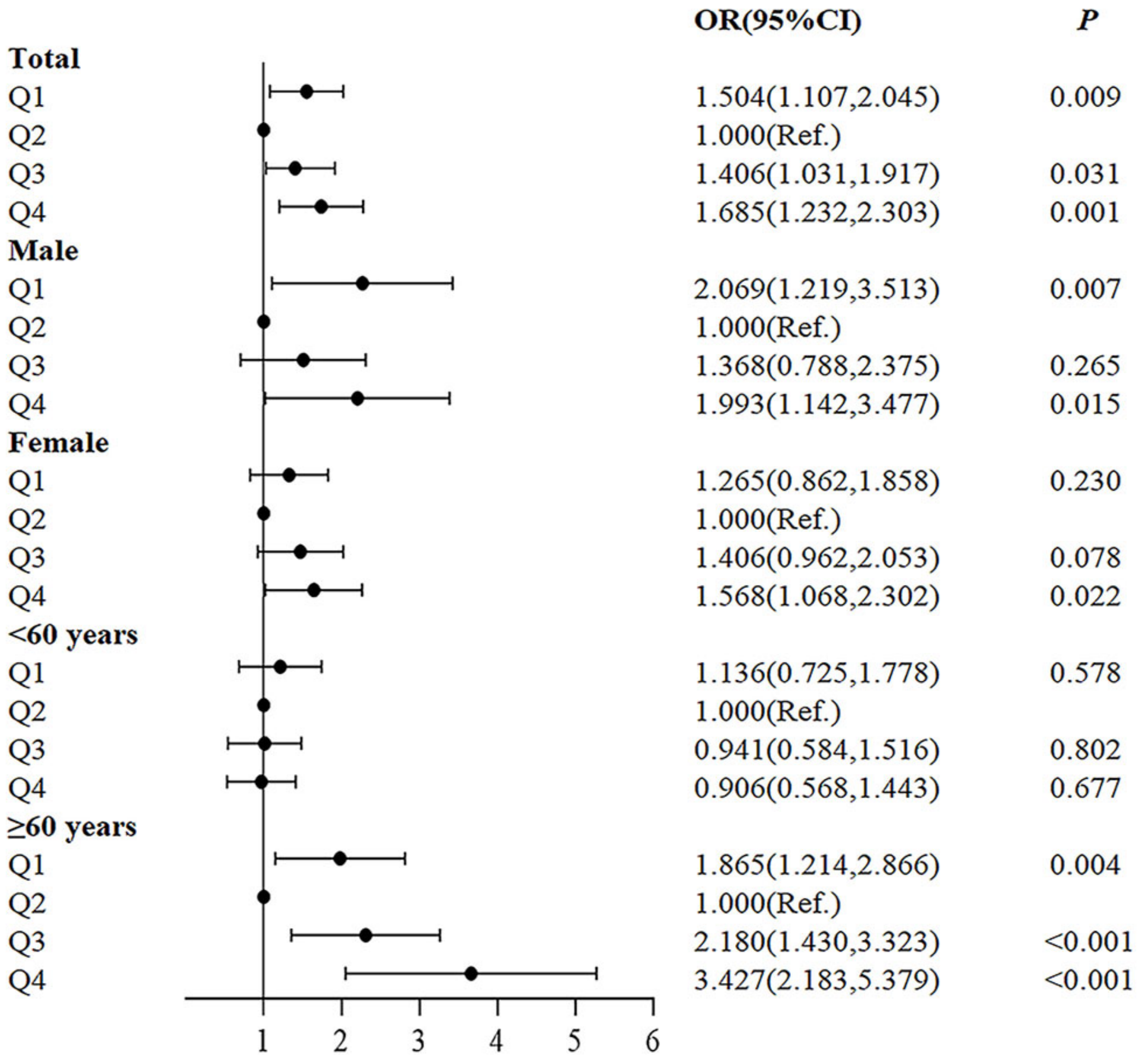

Supplementary figure 1. Subgroup analyses of the association between TMAO concentration levels and MCI. TMAO concentration levels: Q1(< 118.11), Q2(118.11-162.40), Q3(162.41-270.17), Q4(> 270.17), μmol/L. Adjusted, if not stratified, for age, sex, BMI, smoking, drinking, education level, SBP, DBP, fasting glucose, triglyceride, and total cholesterol. MCI, mild cognitive impairment; ORs, odds ratio; CIs, confidence interval.

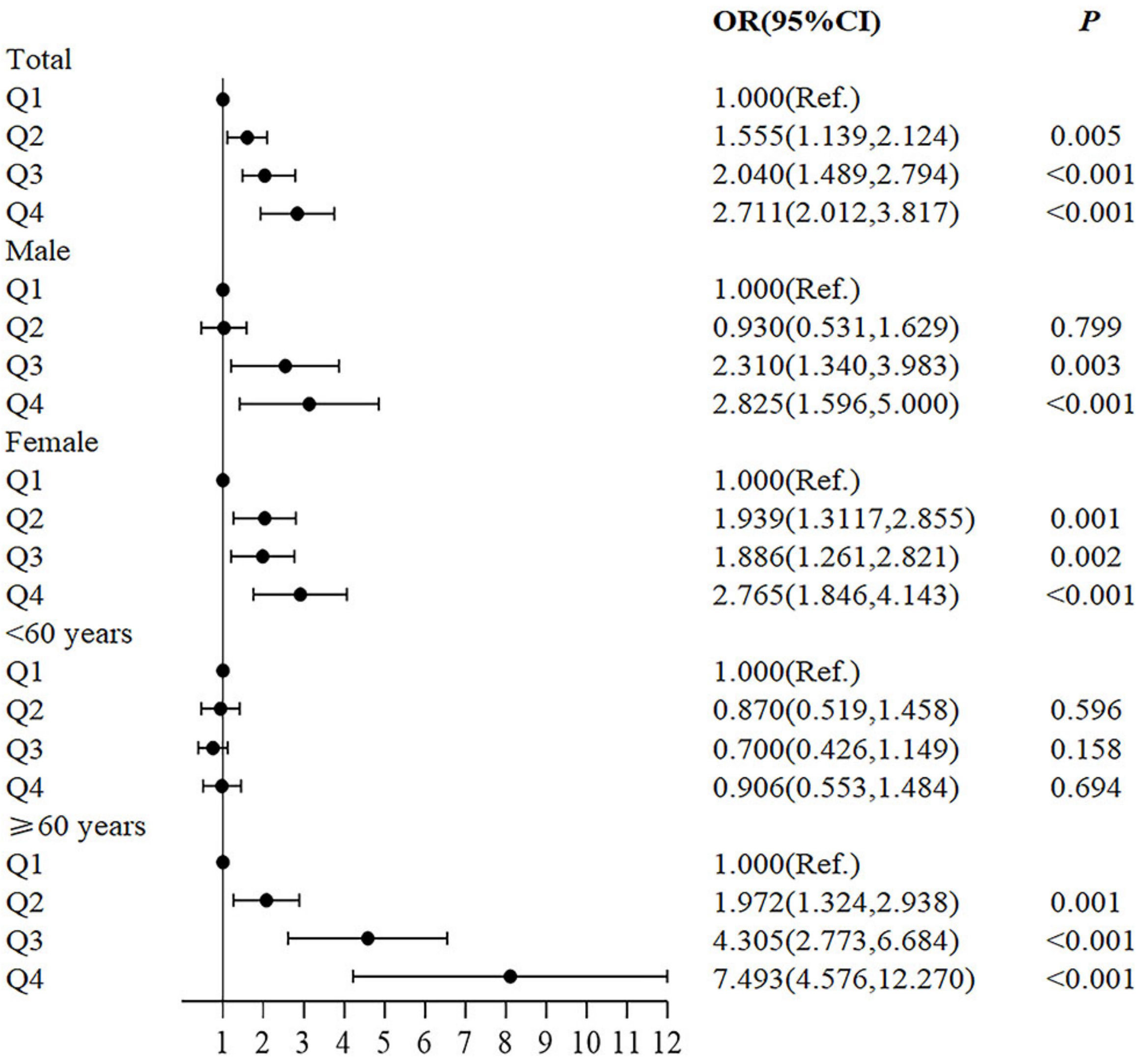

Supplementary figure 2. Subgroup analyses of the association between Choline concentration levels and MCI. Choline concentration levels: Q1(< 118.11), Q2(118.11-162.40), Q3(162.41-270.17), Q4(> 270.17), µmol/L. Adjusted, if not stratified, for age, sex, BMI, smoking, drinking, education level, SBP, DBP, fasting glucose, triglyceride, and total cholesterol. MCI, mild cognitive impairment; ORs, odds ratio; CIs, confidence interval.

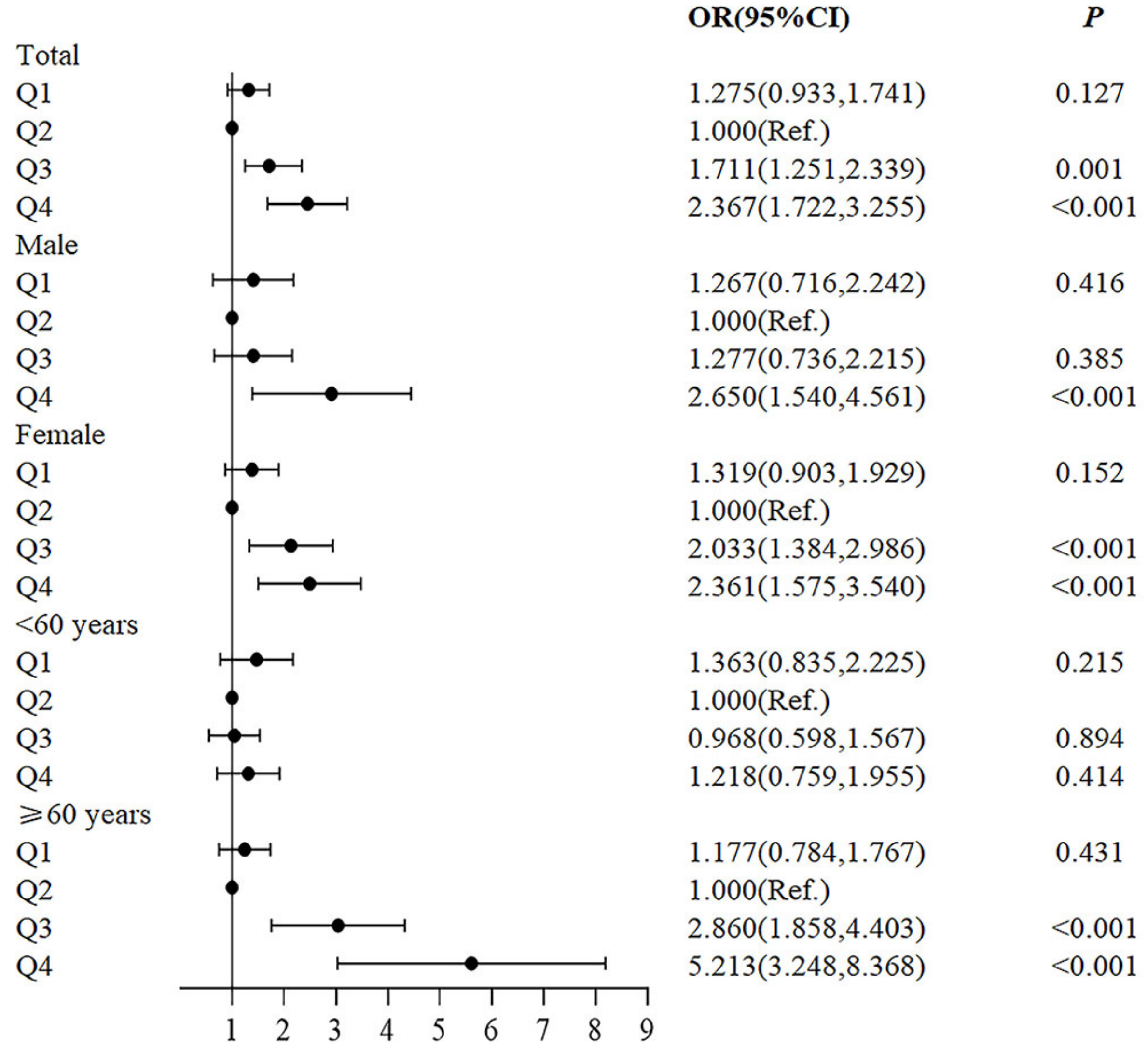

Supplementary figure 3. Subgroup analyses of the association between Betaine concentration levels and MCI. Betaine concentration levels: Q1(< 69.24), Q2(69.24-92.50), Q3(92.51-119.67), Q4(> 119.67), μmol/L. Adjusted, if not stratified, for age, sex, BMI, smoking, drinking, education level, SBP, DBP, fasting glucose, triglyceride, and total cholesterol. MCI, mild cognitive impairment; ORs, odds ratio; CIs, confidence interval.

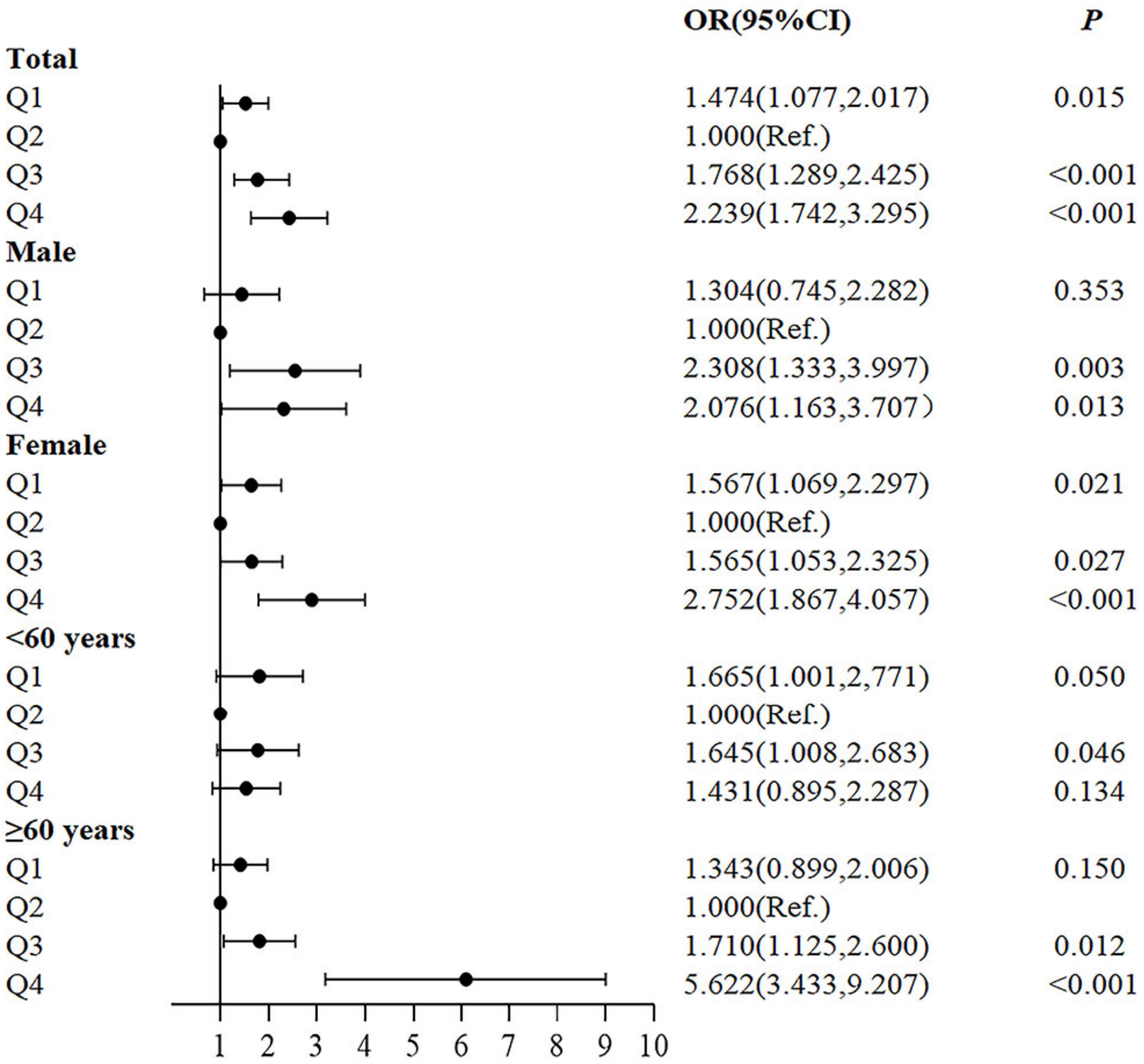

Supplementary figure 4. Subgroup analyses of the association between Carnitine concentration levels and MCI. Carnitine concentration levels: Q1(< 40.09), Q2(40.09-51.18), Q3(51.19-70.90), Q4(> 70.90), μmol/L. Adjusted, if not stratified, for age, sex, BMI, smoking, drinking, education level, SBP, DBP, fasting glucose, triglyceride, and total cholesterol. MCI, mild cognitive impairment; ORs, odds ratio; CIs, confidence interval.
